# Clinical outcomes and prognostic factors in patients with breast diffuse large B cell lymphoma; Consortium for Improving Survival of Lymphoma (CISL) study

**DOI:** 10.1186/1471-2407-10-321

**Published:** 2010-06-22

**Authors:** Ho-Young Yhim, Hye Jin Kang, Yoon Hee Choi, Seok Jin Kim, Won Seog Kim, Yee Soo Chae, Jin Seok Kim, Chul Won Choi, Sung Yong Oh, Hyeon Seok Eom, Jeong-A Kim, Jae Hoon Lee, Jong-Ho Won, Hyeok Shim, Je-Jung Lee, Hwa Jung Sung, Hyo Jung Kim, Dae Ho Lee, Cheolwon Suh, Jae-Yong Kwak

**Affiliations:** 1 Department of Internal Medicine, Chonbuk National University Medical School, Jeonju, Korea; 2 Korea Cancer Center Hospital, Seoul, Korea; 3 Samsung Medical Center, Sungkyunkwan University School of Medicine, Seoul, Korea; 4 Kyungpook National University School of Medicine, Daegu, Korea; 5 Yonsei University College of Medicine, Seoul, Korea; 6 Korea University Medical Center, Guro Hospital, Seoul, Korea; 7 Dong-A University College of Medicine, Busan, Korea; 8 National Cancer Center of Korea, Goyang, Korea; 9 Catholic University of Korea College of Medicine, Seoul, Korea; 10 Gachon University Gil Hospital, Incheon, Korea; 11 Soonchunhyang University College of Medicine, Seoul, Korea; 12 Wonkwang University School of Medicine, Iksan, Korea; 13 Chonnam National University Medical School, Jeollanam-do, Korea; 14 Korea University Medical Center, Ansan Hospital, Ansan, Korea; 15 Hallym University Medical Center and Hallym University College of Medicine, Anyang, Korea; 16 Asan Medical Center, University of Ulsan College of Medicine, Seoul, Korea

## Abstract

**Background:**

The breast is a rare extranodal site of non-Hodgkin lymphoma, and primary breast lymphoma (PBL) has been arbitrarily defined as disease localized to one or both breasts with or without regional lymph nodes involvement. The aim of this study was to evaluate the clinical outcomes in patients with diffuse large B cell lymphoma (DLBCL) and breast involvement, and to find the criteria of PBL reflecting the outcome and prognosis.

**Methods:**

We retrospectively analyzed data from 68 patients, newly diagnosed with DLBCL and breast involvement at 16 Korean institutions between January 1994 and June 2009.

**Results:**

Median age at diagnosis was 48 years (range, 20-83 years). Forty-three (63.2%) patients were PBL according to previous arbitrary criteria, sixteen (23.5%) patients were high-intermediate to high risk of international prognostic index. The patients with one extranodal disease in the breast (OED) with or without nodal disease were 49 (72.1%), and those with multiple extranodal disease (MED) were 19 (27.9%). During median follow-up of 41.5 months (range, 2.4-186.0 months), estimated 5-year progression-free survival (PFS) was 53.7 ± 7.6%, and overall survival (OS) was 60.3 ± 7.2%. The 5-year PFS and OS was significantly higher for patients with the OED group than those with the MED group (5-year PFS, 64.9 ± 8.9% vs. 27.5 ± 11.4%, p = 0.001; 5-year OS, 74.3 ± 7.6% vs. 24.5 ± 13.0%, p < 0.001). In multivariate analysis, MED (hazard ratio [HR], 3.61; 95% confidence interval [CI], 1.07-12.2) and fewer than four cycles of systemic chemotherapy with or without local treatments (HR, 4.47; 95% CI, 1.54-12.96) were independent prognostic factors for worse OS. Twenty-five (36.8%) patients experienced progression, and the cumulative incidence of progression in multiple extranodal sites or other than breasts and central nervous system was significantly different between the OED group and the MED group (5-year cumulative incidence, 9.7 ± 5.4% vs. 49.0 ± 15.1%, p = 0.001).

**Conclusions:**

Our results show that the patients included in OED group, reflecting different treatment outcome, prognosis and pattern of progression, should be considered as PBL in the future trial. Further studies are warranted to validate our suggested criteria.

## Background

Approximately one-third of non-Hodgkin lymphoma (NHL) arises primarily from sites other than lymph nodes; for this reason, they are usually named as primary extranodal lymphoma [1]. The definition of primary extranodal lymphoma remains controversial, especially in patients where both nodal and extranodal sites are involved. In 1961, a strict definition for primary extranodal lymphoma was first proposed by Dawson *et al. *[2] for primary gastrointestinal lymphoma, in which the primary disease was confined to the gastrointestinal tract with involvement of no more than regional lymph nodes. Since then, some authors have suggested that a strict definition of primary extranodal lymphoma, which excluded patients with distant disease from the primary extranodal site, may present an incomplete picture of extranodal lymphoma [3-5]. Hence, more liberal criteria for primary gastrointestinal lymphoma were proposed by other investigators. Lewin *et al. *[3] described a series of patients with primary gastrointestinal lymphoma, which included patients with contiguous other organ involvement and distant nodal involvement as well as the gastrointestinal tract, provided that the extranodal disease was just the presenting site and constituted the predominant disease bulk [6].

Breast lymphoma has been defined as lymphoma involving the breast and historically has been classified into primary breast lymphoma (PBL) and secondary breast lymphoma (SBL). The criteria for PBL have been a controversial, similar to those of other extranodal lymphomas. In 1972, Wiseman and Liao first proposed criteria for PBL as follows: (1) adequate pathologic specimens; (2) mammary tissue and lymphomatous infiltrate in close association; (3) no prior diagnosis of an extramammary lymphoma; and (4) no evidence of concurrent widespread disease except ipsilateral axillary lymph nodes [7]. All patients not meeting these four criteria were considered to have SBL. According to these criteria, all patients with PBL had localized disease with or without regional lymph node involvement.

This strict definition for PBL has been adopted in most subsequent clinical studies or case reports for this rare extranodal lymphoma. However, the clinical study that provided data in developing the defining criteria had several limitations. The study included a total of 31 patients and evaluated only 16 patients with PBL. This, we thought, was an extremely small number of patients to define criteria for classification. In addition, differences in clinical parameters and treatment outcomes between PBL and SBL were not described. As mentioned above, some investigators asserted that these strict criteria did not reflect the biologic behavior of the disease, rather only representing a localized manifestation of a different primary extranodal lymphoma [3-5]. In fact, the traditional strict criteria adopted the pattern of spread that was seen in epithelial breast tumors, from the breast to the regional lymph nodes, even though the pattern of spread in NHL might be different from that of epithelial breast tumors. These findings strongly suggest that the traditional strict criteria for breast lymphoma are not based on scientific considerations about the tumor, but on arbitrary decisions. Nevertheless, until recently, there has been a lack of research investigating more scientific criteria that reflect differences in treatment outcomes and prognosis in patients with NHL and breast involvement.

Therefore, we conducted this retrospective study to investigate scientific classification criteria of primary and secondary breast diffuse large B cell lymphoma (DLBCL) that would reflect treatment outcomes and prognosis. We also evaluated the treatment outcomes of patients with PBL according to the new suggested criteria in terms of progression-free survival (PFS) and OS.

## Methods

### Patients

We consecutively enrolled 68 patients who were newly diagnosed with DLBCL with breast involvement at 16 Korean institutions between January 1994 and June 2009. The two main eligibility criteria included: (1) The histologic diagnosis of DLBCL. The histologic subtypes were classified according to the World Health Organization (WHO) classification, but the Revised European-American Lymphoma (REAL) classification was used before the WHO classification was published in 2001. (2) Documentation of involvement of one or both breasts by the lymphoma, by histology or imaging modalities such as computed tomography or positron emission tomography if other sites were biopsied as needed. Patients presenting with recurrent lymphoma in the breast following prior treatment were excluded. According to the traditional strict criteria, PBL was defined as disease localized to one or both breasts with or without regional lymph nodes involvement (ipsilateral axillary and/or supraclavicular lymph nodes), and SBL was defined as disease with systemic lymph nodes and/or other extranodal organ involvement as well as involvement of one or both breasts. Mediastinal and cervical lymph nodes were not regarded as regional lymph nodes. Bone marrow was considered an extranodal site. By the number of involved extranodal disease, one extranodal disease in the breast (OED) was defined as disease with only breast involvement with or without nodal disease, which included distant systemic nodal disease as well as regional nodal disease. Multiple extranodal disease (MED) was defined as disease with multiple extranodal organ involvement including one or both breasts regardless of any involvement of lymph nodes (Table 1). Namely, the MED group included patients who had two or more extranodal disease sites including one or both breasts. The patients were staged according to the Ann Arbor staging system, and the International Prognostic Index (IPI) was determined for prognosis. Other clinical characteristics, including demographics, laboratory profiles, disease-related profiles, treatment, and treatment outcomes were collected using study-specific case record forms. This study protocol was approved by the Institutional Review Board (IRB) from each participating institution.

**Table 1:** Classification of breast lymphoma according to the number of involved extranodal organs

	Definition
OED	Isolated extranodal organ (i.e. breast) involvement with or without nodal disease, which included distant systemic nodal disease as well as regional nodal disease.
	
MED	Multiple extranodal organ involvement including one or both breasts regardless of any involvement of lymph nodes

Abbreviations: OED, one exgtranodal disease in the breast; MED, multiple extranodal disease.

### Criteria for Treatment Outcomes

Tumor response was evaluated using the International Working group Criteria (IWC) [8]. PFS was defined as the time from the first day of treatment to the date on which progressive disease was first documented or to the date of last follow-up. OS was calculated as the time from the first day of treatment to the date of death or the date of last follow-up.

### Statistical Analysis

Descriptive statistics were summarized as frequencies and percentages for categorical variables and as median and range for continuous variables. The comparisons of clinical variables between the OED group and MED group were made using χ^2 ^test or Fisher's exact test for categorical variables, and Mann-Whitney test for continuous variables. The distribution of PFS and OS was estimated using the Kaplan-Meier method, and comparisons between groups were made using log-rank tests. Multivariate analysis was carried out using the Cox proportional hazards models, reported with hazard ratio (HR) and 95% confidence interval (CI), and a significance level of 0.05 was used for covariate entry. The cumulative incidence of first progression according to the predominant sites was estimated using the Kalbfleisch and Prentice method [9]. Two-tailed p value of <0.05 was considered statistically significant. SPSS for windows, version 12.0 (SPSS Inc., Chicago, IL, USA), was used for all statistical analyses.

## Results

### Demographic and Clinical Characteristics

Demographic and clinical characteristics of these 68 patients are summarized in Table 2. The median age at diagnosis was 48 years (range, 20-83 years) and all patients were female. Forty-five (66.2%) patients were localized stages (I/II), 26 (38.2%) patients elevated level of lactate dehydrogenase (LDH) and 5 (7.4%) patients had B symptoms. Sixteen (23.5%) patients were classified as high-intermediate to high risk according to the IPI. Two (2.9%) patients were bulky at diagnosis. Sixty-seven (98.5%) patients were treated with systemic chemotherapy, 66 (97.1%) treated with anthracycline-based regimens. Fifty-five (80.9%) patients were treated with four or more than four cycles of systemic chemotherapy with our without any local treatment modalities such as surgery or radiotherapy. Any surgery or radiotherapy to the breasts was performed in 23 (33.8%) and 21 (30.9%) patients, respectively. Forty-two patients (61.8%) were received immunochemotherapy with rituximab.

**Table 2:** Demographics and clinical characteristics of 68 patients

Characteristics	Total (n = 68)	OED (n = 49)	MED (n = 19)	P value
Age at diagnosis, year				
Median	48	45	52	0.589
Range	20-83	20-73	20-83	
Sex				
Male	0	0	0	
Female	68	49	19	
Duration of follow-up, months				
Median	41.5	46.3	34.7	0.176
Range	2.4-186.0	2.4-186.0	2.5-123.5	
Ann Arbor stage				
I-II	45 (66.2%)	45 (91.8%)	0 (0%)	<0.001
III-IV	23 (33.8%)	4 (8.2%)	19 (100%)	
B symptoms				
Absent	63 (92.6%)	47 (95.9%)	16 (84.2%)	0.129
Present	5 (7.4%)	2 (4.1%)	3 (15.8%)	
ECOG performance status				
0-1	63 (92.6%)	47 (95.9%)	16 (84.2%)	0.129
2-3	5 (7.4%)	2 (4.1%)	3 (15.8%)	
LDH level				
Normal	36 (52.9%)	29 (59.2%)	7 (36.8%)	0.098
Elevated	26 (38.2%)	16 (32.7%)	10 (52.6%)	
Unknown	6 (8.8%)	4 (8.1%)	2 (10.5%)	
IPI				
Low to low-intermediate	50 (73.5%)	44 (89.8%)	6 (31.6%)	<0.001
High-intermediate to high	16 (23.5%)	3 (6.1%)	13 (68.4%)	
Unknown	2 (2.9%)	2 (4.1%)	0 (0%)	
Traditional strict criteria				
Traditional PBL	43 (63.2%)	43 (87.8%)	0 (0%)	N/A
Traditional SBL	25 (36.8%)	6 (12.2%)	19 (100%)	
Bulky disease				
No	66 (97.1%)	48 (98.0%)	18 (94.7%)	0.484
Yes	2 (2.9%)	1 (2.0%)	1 (5.3%)	
Systemic chemotherapy				
No	1 (1.5%)	0 (0%)	1 (5.3%)	0.279
Yes	67 (98.5%)	49 (100%)	18 (94.7%)	
Surgery of the breast				
No	45 (66.2%)	28 (57.1%)	17 (89.5%)	0.012
Yes	23 (33.8%)	21 (42.9%)	2 (10.5%)	
Radiotherapy to the breast				
No	47 (69.1%)	29 (59.2%)	18 (94.7%)	0.004
Yes	21 (30.9%)	20 (40.8%)	1 (5.3%)	
Treatment strategy				
<4 cycles ± local modalities	12 (17.6%)	8 (16.3%)	4 (21.1%)	0.577
≥4 cycles ± local modalities	55 (80.9%)	41 (83.7%)	14 (73.6%)	
No treatment	1 (1.5%)	0 (0%)	1 (5.3%)	
Rituximab use				
No	26 (38.2%)	23 (46.9%)	3 (15.8%)	0.025
Yes	42 (61.8%)	26 (53.1%)	16 (84.2%)	
Enroll period				
1994-2002	21 (30.9%)	19 (38.8%)	2 (10.5%)	0.039
2003-2009	47 (69.1%)	30 (61.2%)	17 (89.5%)	
Extranodal involvement				
Breast	68 (100%)	49 (100%)	19 (100%)	N/A
Liver	6 (8.8%)	0	6 (31.6%)	
Bone	6 (8.8%)	0	6 (31.6%)	
Lung	4 (5.9%)	0	4 (21.1%)	
Bone marrow	4 (5.9%)	0	4 (21.1%)	
Stomach	3 (4.4%)	0	3 (15.8%)	
Nasal cavity	3 (4.4%)	0	3 (15.8%)	
Central nervous system	3 (4.4%)	0	3 (15.8%)	
Adrenal gland	2 (2.9%)	0	2 (10.5%)	
Pancreas	2 (2.9%)	0	2 (10.5%)	
Thyroid	1 (1.5%)	0	1 (5.3%)	
Ovary	1 (1.5%)	0	1 (5.3%)	

Values are expressed as the number of patients (%) unless stated otherwise. Abbreviations: OED, one exgtranodal disease in the breast; MED, multiple extranodal disease; PBL, primary breast lymphoma; ECOG, Eastern Cooperative Oncology Group; LDH, lactate dehydrogenase; IPI, international prognostic index; SBL, secondary breast lymphoma; BM, bone marrow.

According to the number of involved extranodal organ, the OED group included 49 (72.1%) patients, and the MED group included 19 (27.9%) patients. By definition, all patients in the MED group were Ann Arbor stage IV disease and had at least two clinical risk factors for IPI, more than one site of extranodal disease and stage. The median number of involved extranodal organ in MED group was 3 (range, 2-5). The most commonly involved extranodal organs except breast were liver and bone (n = 6, 31.6%, respectively), which were followed by lung (n = 4), bone marrow (n = 4), stomach (n = 3), nasal cavity (n = 3), central nervous system (n = 3), adrenal gland (n = 2), pancreas (n = 2), thyroid (n = 1) and ovary (n = 1). Patients in the OED group were Ann Arbor stage I to III, and all PBL patients according to the traditional strict criteria were included in the OED group. As a result, more patients in the OED group had low to low-intermediate IPI than in the MED group (89.8% *vs*. 31.6%, p < 0.001). When we compared the classification according to the number of involved extranodal organ with the traditional strict criteria, six patients diagnosed with SBL by the traditional strict criteria included into the OED group. Of the six patients, four patients were stage III, and two patients were stage II; five patients had low to low-intermediate of IPI, and one patient had high to high-intermediate IPI. Treatment with fewer than four cycles of systemic chemotherapy following surgery or followed by radiotherapy did not differ between the two groups. Among 21 patients treated with surgery in the OED group, modified radical mastectomy was performed in 13 (61.9%) patients and lumpectomy with or without axillary node dissection, in 8 (38.1%) patients. The MED group received rituximab more frequently than OED group (84.2% vs. 53.1%, p = 0.025) (Table 2).

### Treatment Outcomes

Response data for initial treatment were available for 65 (95.6%) patients. Two patients could not be evaluated for response because of systemic chemotherapy at the time of analysis, and one patient could not be determined due to treatment-related septic death before response evaluation. Complete response occurred in 54 (83.1%) patients, and partial response, in six (9.2%) patients, for an overall response of 92.3% with initial treatment. Overall response was significantly higher in the OED group (97.9%) than in the MED group (76.5%, p = 0.015).

With median follow-up of 41.5 months (range, 2.4-186.0 months), estimated 5-year PFS was 53.7 ± 7.6% and OS was 60.3 ± 7.2%. Univariate analysis of factors influencing PFS and OS is presented in Table 3. In univariate analysis for PFS, Ann Arbor stage of III or IV, ECOG performance status of 2 or 3, elevated levels of LDH, high-intermediate to high IPI, the SBL group according to traditional strict criteria, the MED group and fewer than four cycles of systemic chemotherapy with or without local treatment were significantly associated with lower PFS (Table 3). In multivariate analysis for PFS, independent prognostic factors for lower PFS included the MED group (HR, 4.11; 95% CI, 1.28-13.2), a high-intermediate to high IPI (HR, 3.35; 95% CI, 1.05-10.72) and fewer than four cycles of systemic chemotherapy with or without local treatment (HR, 3.90; 95% CI, 1.30-11.70) (Table 4).

**Table 3:** Univariate analysis of prognostic factors for progression-free survival and overall survival

Variables	No. of patients	5-year PFS (95% CI)	P value	5-year OS (95% CI)	P value
Age at diagnosis (years)					
<60	55	54.0 ± 8.1	0.943	61.6 ± 7.8	0.616
≥60	13	58.2 ± 17.0		57.3 ± 17.0	
Ann Arbor stage					
I-II	45	61.3 ± 9.2	0.038	70.7 ± 8.0	0.010
III-IV	23	38.5 ± 11.9		35.1 ± 13.8	
B symptoms					
No	63	54.3 ± 7.8	0.288	61.8 ± 7.4	0.088
Yes	5	50.0 ± 25.0		50.0 ± 25.0	
ECOG performance status					
0-1	63	55.8 ± 7.9	0.016	63.4 ± 7.4	0.015
2-3	5	40.0 ± 21.9		25.0 ± 21.7	
LDH level					
Normal	36	68.3 ± 11.2	0.007	71.0 ± 8.9	0.049
Elevated	26	36.1 ± 11.8		40.1 ± 12.3	
No. of involved extranodal organ					
OED	49	64.9 ± 8.9	0.001	74.3 ± 7.6	<0.001
MED	19	27.5 ± 11.4		24.5 ± 13.0	
IPI					
Low to low-intermediate	50	63.2 ± 9.1	<0.001	69.2 ± 8.1	0.001
High-intermediate to high	16	25.0 ± 12.3		23.6 ± 13.7	
Traditional strict criteria					
Traditional PBL	43	89.4 ± 5.9	0.008	72.6 ± 8.0	0.008
Traditional SBL	25	61.7 ± 12.8		33.3 ± 13.4	
Radiotherapy to the breast					
No	47	58.4 ± 8.5	0.777	56.6 ± 9.4	0.477
Yes	21	51.8 ± 12.0		66.7 ± 11.3	
Surgery of breast					
No	45	53.4 ± 9.6	0.839	58.6 ± 9.7	0.582
Yes	23	57.2 ± 11.5		64.1 ± 10.9	
Treatment strategy					
<4cycles ± any local modalities	12	28.0 ± 16.4	<0.001	19.3 ± 16.2	<0.001
≥4cycles ± any local modalities	55	58.0 ± 8.3		66.2 ± 7.7	
Rituximab use					
No	26	56.8 ± 11.3	0.366	60.6 ± 10.3	0.975
Yes	42	53.5 ± 9.5		60.3 ± 10.3	

Abbreviations: PFS, progression-free survival; OS, overall survival; CI, confidence interval; ECOG Eastern Cooperative Oncology Group; LDH, lactate dehydrogenase; PBL, primary breast lymphoma; OED, one extranodal disease in the breast; MED, multiple extranodal disease; IPI, international prognostic index; SBL, secondary breast lymphoma.

**Table 4:** Multivariate analysis of prognostic factors for progression-free survival and overall survival

Variables	Hazard ratio	95% CI	P value
PFS			

No. of involved extranodal organ			
OED	1		
MED	4.11	1.28-13.2	0.017
IPI			
Low to low-intermediate	1		
High-intermediate to high	3.35	1.05-10.72	0.042
Treatment strategy			
≥4cycles ± any local modalities	1		
<4cycles ± any local modalities	3.90	1.30-11.70	0.015

OS			

No. of involved extranodal organ			
OED	1		
MED	3.61	1.07-12.20	0.039
IPI			
Low to low-intermediate	1		
High-intermediate to high	3.58	0.99-12.98	0.052
Treatment strategy			
≥4cycles ± any local modalities	1		
<4cycles ± any local modalities	4.47	1.54-12.96	0.006

Abbreviation: PFS, progression-free survival; OS, overall survival; CI, confidence interval; OED, one extranodal disease in the breast; MED, multiple extranodal disease; IPI, international prognostic index.

In univariate analysis for OS, Ann Arbor stage of III or IV, ECOG performance status of 2 or 3, elevated levels of LDH, high-intermediate to high IPI, the SBL group according to traditional strict criteria, the MED group, and fewer than four cycles of systemic chemotherapy with or without local treatment were significantly associated with lower OS (Table 3). In multivariate analysis for OS, the MED group (HR, 3.61; 95% CI, 1.07-12.2) and fewer than four cycles of systemic chemotherapy with or without local treatment (HR, 4.47; 95% CI, 1.54-12.96) were independent prognostic factors for lower OS (Table 4). Other risk factors of earlier death included in multivariate model were IPI (high-intermediate to high) and traditional strict criteria (SBL). However, these variables were not independent prognostic factors for OS. Immunochemotherapy with rituximab was not associated with improved PFS or OS.

**Figure 1:**
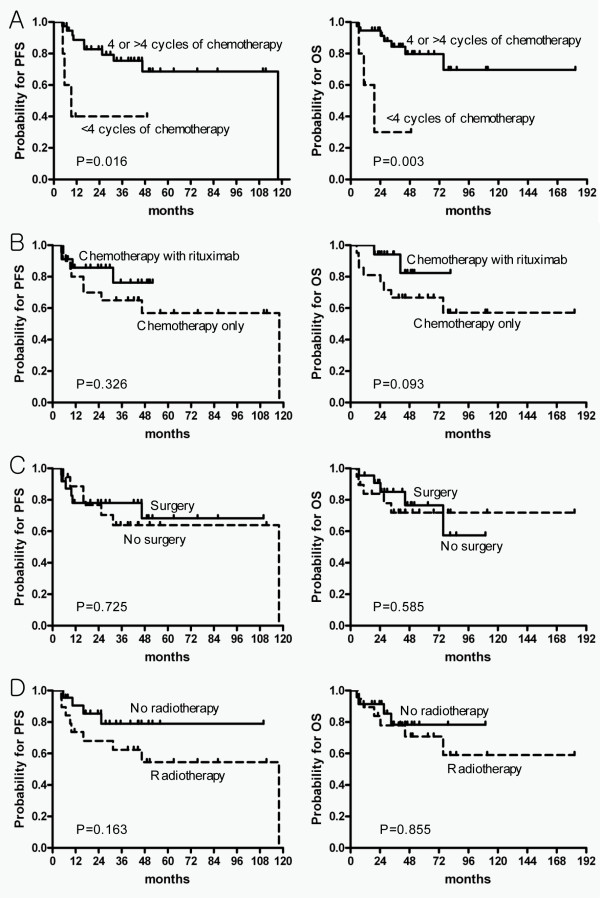
**Kaplan-Meier survival curves of PFS (left) and OS (right) by each type of treatment in the OED group**. (A) By number of cycles of systemic chemotherapy (fewer than four versus four or more); (B) the use of rituximab; (C) surgery, and (D) radiotherapy. Abbreviations: PFS, progression-free survival; OS, overall survival; OED, one extranodal disease in the breast.

In the subgroup analysis of the OED group according to the number of involved extranodal organ, the patients who had fewer than four cycles of systemic chemotherapy had significantly worse 5-year PFS and OS, compared with patients with four or more cycles of systemic chemotherapy (5-year PFS, 40% *vs*. 68.6%, p = 0.016; 5-year OS, 30% *vs*. 79.6%, p = 0.003, Figure 1A) However, we found that the use of immunochemotherapy with rituximab, surgery, and radiotherapy were not associated with differences in PFS and OS (Figure 1B, C, D)

### Patterns of Progression

Twenty-five (36.8%) patients experienced progressive disease following first-line treatment, and nine (13.2%) patients had two or more organs involved at first progression. Lymph nodes were a first site of progression in 10 patients, breast in nine, CNS in seven, intestine in three, lung in three, bone marrow in two, and liver, nasal cavity, ovary, adrenal gland each in one patient. Table 5 represents the cumulative incidence of progression at each predominant involvement site. When the cumulative incidence of progression of lymph nodes only, breasts (with or without lymph nodes, with or without CNS) and CNS (with or without lymph nodes, with or without other extranodal disease, but not lymph nodes only and breast) were compared between the OED and MED groups, they were not significantly different, whereas the cumulative incidence of extranodal progression in two or more sites or other than breasts and CNS was significantly higher in the MED group than in the OED group. Breast was the most common site of first progression in the OED group (six patients, 12.2%). And, CNS was a first progression site in five (10.2%) patients among 49 patients in the OED group. Five (10.2%) patients in the OED group were treated with prophylactic intrathecal chemotherapy, and there was no CNS progression in these patients.

**Table 5:** Comparison of cumulative incidence of predominant progression sites between one extranodal disease in the breast and multiple extranodal disease group according to suggested criteria

	Disease classification	**No**.	1-year	3-year	5-year	P value
LNs only	OED	1	2.7 ± 2.7	2.7 ± 2.7	2.7 ± 2.7	0.062
	MED	2	7.7 ± 7.4	10.2 ± 8.2	10.2 ± 8.2	

Breast (±LNs, ±CNS)	OED	6	7.8 ± 4.3	11.9 ± 5.8	16.6 ± 7.1	0.902
	MED	2	13.7 ± 9.2	13.7 ± 9.2	13.7 ± 9.2	

CNS (±LNs, ± other extranodal diseases, but not LNs only and	OED	4	5.5 ± 3.8	8.7 ± 4.8	12.3 ± 8.5	0.411
breast)	MED	2	12.5 ± 8.3	12.5 ± 8.3	12.5 ± 8.3	

Extranodal diseases (±LNs, ≥2 or other than	OED	3	2.9 ± 2.8	9.7 ± 5.4	9.7 ± 5.4	0.001
breast and CNS)	MED	6	38.7 ± 14.2	49.0 ± 15.1	49.0 ± 15.1	

Values are expressed as cumulative incidence (%) ± standard error unless stated otherwise. Abbreviations: OED, one extranodal disease in the breast; MED, multiple extranodal disease; LNs, lymph nodes; CNS, central nervous system.

## Discussion

PBL is a rare clinical presentation of NHL with a prevalence of 0.04% to 1.1% of all breast tumors and 1.7% to 2.2% of all extranodal NHL [10]. However, the definition of PBL is still unclear. Criteria for PBL were first proposed by Wiseman and Liao in 1972 [7], which were ambiguous and arbitrary criteria that did not consider the biologic behavior of NHL and were not validated as to prognostic significance.

In this study, we evaluated clinical parameters, treatment outcomes, prognostic factors, and patterns of progression in patients with DLBCL of the breast, and found the classification by the number of involved extranodal organ, OED group and MED group, could reflect the differences. According to the suggested classification criteria, all patients in the MED group were Ann Arbor stage IV, whereas patients in the OED group were Ann Arbor stages I to III. As a result, more patients in the OED group had low to low-intermediate IPI than in the MED group. When we compared traditional strict criteria with our suggested criteria, 5-year PFS in the MED group were markedly lower than in the SBL group (5-yr PFS, 27.5 ± 11.4% *vs*. 61.7 ± 12.8%). This may indicate that some proportion of patients in the traditional SBL group have a relatively favorable treatment outcome and probably a different biology than those who had MED. Furthermore, although both the criteria by the number of involved extranodal organ and the traditional strict criteria showed statistically significant differences by univariate analysis, only the criteria according to the number of involved extranodal organ were an independent factor for predicting PFS and OS by multivariate analysis. Thus, the classification according to the number of involved extranodal organ had better discriminatory power for predicting survival by separating the patients who had a favorable outcome in the SBL group without conflating the different clinical characteristics and treatment outcomes between PBL and SBL seen in the traditional strict criteria. Moreover, comparing the predominant sites of progression in the OED and MED groups, interesting features were observed. Patients in the MED group were significantly more likely to have multiple extranodal organ recurrence, compared with the OED group. On the other hand, breast (six patients, 12.2%) was the most common site of progression in the OED group, indicating that the patterns of progression may be different according to the number of extranodal organ. Therefore, the OED group according to the number of involved extranodal organ should be considered as PBL in the subsequent clinical studies, because this could reflect the differences in treatment outcome, prognosis and patterns of progression.

It was an interesting finding that rituximab was not associated with improved outcome in our cohort. It might be related with the fact that the MED group received rituximab more frequently than OED group. In our country, rituximab was available since 2003. From 1994 to 2002, twenty-one patients were consecutively enrolled to this study, and they, of course, were treated without rituximab. Of these patients, 19 patients were the OED group, whilst only 2 patients were the MED group (p = 0.039). This might explain why the MED group received rituximab more frequently than OED group. On the long-term results of pivotal trial about rituximab conducted by the Groupe d'Etude des Lymphomes de l'Adulte (GELA), immunochemotherapy with rituximab showed the significant superiority of OS over chemotherapy alone in low-risk patients according to age-adjusted IPI [11]. However, the difference of OS in high-risk patients was not evident. The MED group, in our analysis, was absolutely poor-risk group because about two-thirds of patients were high-intermediate to high risk according to IPI. Therefore, the lack of impact on OS of rituximab might be associated with fact that rituximab was more frequently used in poor-risk patients.

The prognostic relevance of the IPI in patients with breast lymphoma has been validated in several studies [12,13]. The IPI in our population was also an independent prognostic factor for PFS but not for OS. Our study had relatively short follow-up duration (median 41.5 months). As a result, only 10 (20.4%) patients in the PBL group had died by the time of analysis. Because of this point, further follow-up is needed to evaluate the influence of IPI on OS.

Recently, the International Extranodal Lymphoma Study Group (IELSG) reported the results of the largest international survey to date of 204 patients with primary localized DLBCL of the breast, in which the study adopted traditional strict criteria for PBL [12]. When we compared the survival of our PBL group, which indicated OED group according to the number of involved extranodal organ, with outcomes seen in the IELSG survey, 5-year PFS (64.9%) and OS (74.3%) in our cohort were superior to those in IELSG survey (54% and 63%, respectively), even though direct comparison is difficult because of the different definitions of PBL. The relatively good survival in our PBL group may reflect different patient characteristics, compared with those from the IELSG survey. The median age in our cohort (45 years) was younger than that in the IELSG survey (64 years), and the proportion of patients with low to low-intermediate IPI in our cohort (89.8%) was higher than in the IELSG cohort (78%). The patients in our cohort may have more favorable prognostic indices, compared with those in the IELSG survey, and this was associated with more favorable survival even though the patients with more advanced stages were included in our PBL group.

Our study also confirmed the previously reported high rate of extranodal recurrence in the PBL [10,12-17]. The sites of progression in the OED group were mainly extranodal. Only one patient had progression in lymph nodes without extranodal disease. One of the notable findings in our analysis was relatively high risk of CNS progression in the OED group, even with relatively short follow-up duration. In our analysis, CNS progression occurred in 10.2% of patients in the PBL group. Although considerably lower CNS progression (5%) occurred in the IELSG survey [12], other PBL series consistently showed more than 10% of CNS progression [10,13-15,17]. Furthermore, other extranodal primary DLBCL, such as primary testicular DLBCL, has a high risk of CNS progression of approximately 15% up to10 years [18]. Concerns about late CNS progression in patients with extranodal lymphoma, in contrast to early relapse within 1 year in patients with aggressive nodal lymphoma, were raised in this primary testicular series [18]. Although it is not clear whether late CNS progression is a unique characteristic of primary testicular lymphoma or a common feature of extranodal lymphoma, we cannot exclude the possibility of further CNS progression in our cohort. Additionally, it is an interesting finding that CNS progression did not occur in the five patients who received prophylactic intrathecal chemotherapy. Although the value of prophylactic intrathecal chemotherapy could not be evaluated in this analysis because of the small number of patients, we thought this may be a meaningful finding when we considered that no CNS progression occurred in a subgroup of patients who received prophylactic intrathecal chemotherapy in the IELSG survey as well [12]. Thus, further studies are needed to provide solid evidence to support the use of prophylactic intrathecal chemotherapy in patients with PBL.

There is still no universal standard treatment of PBL. Most studies agree that the combination of limited surgery, anthracycline-based systemic chemotherapy, and radiotherapy is the best therapeutic option in patients with PBL [12,13,17]. Interestingly, more than four cycles of anthracycline-based systemic chemotherapy was associated with improved PFS and OS in the PBL group, whereas radiation therapy was not associated with improved PFS and OS. This observation may be quite different from previous PBL series, which reported that radiation therapy was associated with survival benefit [12,13]. However, our results about radiation therapy should be interpreted with caution because a high proportion of patients (61.9%) in PBL group were treated with modified radical mastectomy. Several studies reported that survival was not improved by surgery in patients with PBL [12,16,19]. Furthermore, the IELSG reported that radical mastectomy, which caused delayed initiation of systemic chemotherapy, was associated with poorer survival [12]. In our study, the proportion of patients treated with modified radical mastectomy was more than twice that of the IELSG survey (30%), and this factor might offset the merits of radiation therapy. Also, an abbreviated course of systemic chemotherapy as well as extensive surgery might influence the poorer survival in our analysis. A randomized trial, comparing a full course of cyclophosphamide, doxorubicin, vincristine, and prednisolone (CHOP) alone with an abbreviated course of CHOP followed by involved-field radiotherapy (IFRT) in patients with nonbulky, stage I-II, nodal DLBCL, was conducted by the Southwest Oncology Group, and the abbreviated course of CHOP plus radiotherapy was superior to full course CHOP alone, with median follow-up of 4.4 years [20]. However, an update of that study with longer follow-up showed convergence of survival curves as a result of an excess of relapses and deaths from lymphoma in the group given the abbreviated course of CHOP plus IFRT [21]. The authors explained that these results might be associated with the inclusion of patients with adverse prognostic factors, such as elevated LDH levels seen in 20% of patients. Thus, the Southwest Oncology Group study emphasized the significance of systemic chemotherapy in patients with adverse prognostic factors. Our analysis included about one-third of patients with elevated levels of LDH in the OED group, and it is likely that an abbreviated course of systemic chemotherapy negatively influenced the outcome in these patients.

Generally, the addition of rituximab to chemotherapy has improved of survival in patients with aggressive nodal DLBCL [22,23]. However, the impact on survival of rituximab has never been studied in patients with PBL. Persky *et al. *[24] reported that the addition of rituximab to three cycles of CHOP plus IFRT in patients with high-risk, limited-stage DLBCL, resulted in modest improvement of PFS and OS compared with historical cohort. In that study, high-risk patients were defined as those with at least one adverse risk factor by the stage-modified IPI (nonbulky stage II disease, age >60 years, WHO performance status of 2, or elevated serum LDH). However, this improvement was smaller than observed in advanced disease, and they hypothesized that the modest improvement might be related with a unique biology of limited-stage DLBCL contrast to advanced-stage disease. This finding was meaningful for us to infer the role of rituximab in patients with PBL. More than two-thirds (71.4%) in the OED group of our cohort were high-risk, limited-stage disease. And thus, it was likely that rituximab in patients with PBL might add smaller benefit than as we expected. Furthermore, there was a report suggesting that survival benefits with rituximab were not evident in patients with PBL, in which patients were treated with rituximab and dose-dense chemotherapy (CEOP14) and compared with historical controls [25]. In our analysis of OED group, they did not show a significant PFS difference between patients with chemotherapy plus rituximab and those with chemotherapy alone, but only showed a trend of improved OS for patients treated with chemotherapy plus rituximab. In contrast to nodal, advanced DLBCL, limited role of immunochemotherapy with rituximab would be suggested in this rare extranodal DLBCL. Thus, even though success of rituximab in nodal, advanced-stage DLBCL, further studies are needed for demonstrating the efficacy of rituximab in patients with PBL.

The limitations of the current study include the followings. First, the small number of patients, heterogeneity of the population and short follow-up duration are important limitations of this study. Second, molecular prognostic phenotypes such as germinal center (GC) and activated B cell were not included in this analysis because study protocol approved by IRBs of each center did not require mandatory tissue collection. Recently, some studies have reported that GC phenotype of DLBCL have a significantly better outcome compared to patients with the non-GC phenotype [26,27]. However, other studies suggested that the addition of rituximab to CHOP-like chemotherapy was associated with overcoming the adverse prognostic impact of non-GC phenotype [28,29]. About two-thirds of patients (61.8%) in our analysis were treated with rituximab-containing regimens. Therefore, it was likely that the influence of molecular phenotypes on prognosis would quite be decreased in our cohort. Nonetheless, it is major limitation that the results of molecular phenotypes on this series of patients are not available.

As we stated above, several questions about treatment strategies of PBL were raised in this retrospective analysis. Solid evidence, as we know, should come from the results of randomized trials designed on the basis of findings from retrospective studies. The rarity of the disease, however, makes randomized trials virtually impossible in a single institution or nation. Hence, international collaboration is required to adequately conduct randomized trials. Future trials for PBL should consider adopting the criteria according to the number of involved extranodal organ, and include questions raised in our study, including the role of rituximab, prophylactic intrathecal chemotherapy, and radiation therapy. The results of such trials can provide evidence to define the best treatment strategies for PBL.

## Conclusions

In conclusion, our analysis showed classification criteria for PBL reflecting differences in treatment outcomes, prognosis and patterns of progression, compared with the traditional strict criteria. A full course of anthracycline-containing systemic chemotherapy appeared to be the most important treatment for patients with PBL. However, we also found that extensive radical surgery might be harmful and therefore should be avoided. Nevertheless, extranodal progression, especially in the breast and CNS, is the main reason for treatment failure in patients with PBL, resulting in shorter long-term survival. Further studies of prospective design with international collaboration are warranted to evaluate the role of rituximab, prophylactic intrathecal chemotherapy, and radiation therapy in patients with PBL according to our suggested criteria.

## Competing interests

The authors declare that they have no competing interests.

## Authors' contributions

HYY participated in design of the study, ethical approval, review of clinical data, and drafted the manuscript. JYK and CS participated in the design of this study, coordination of the study, and critically revised the manuscript. HJK and SJK performed the statistical analysis of the study. WSK, YSC, JSK, CWC, OSY, HSE and JAK collected the data, and helped to draft the manuscript and to interpret the results. YHC, JHL, JHW, HS, JJL, HJS, HJK and DHL collected data, and revised manuscript. All authors read and approved the final manuscript.

## Pre-publication history

The pre-publication history for this paper can be accessed here:

http://www.biomedcentral.com/1471-2407/10/321/prepub

## References

[B1] Zucca E Extranodal lymphoma: a reappraisal Ann Oncol 2008 19 Suppl 4 iv77 80 10.1093/annonc/mdn204 18519412

[B2] Dawson IM Cornes JS Morson BC Primary malignant lymphoid tumours of the intestinal tract. Report of 37 cases with a study of factors influencing prognosis Br J Surg 1961 49 80 89 10.1002/bjs.18004921319 13884035

[B3] Lewin KJ Ranchod M Dorfman RF Lymphomas of the gastrointestinal tract: a study of 117 cases presenting with gastrointestinal disease Cancer 1978 42 2 693 707 10.1002/1097-0142(197808)42:2<693::AID-CNCR2820420241>3.0.CO;2-J 354774

[B4] Krol AD le Cessie S Snijder S Kluin-Nelemans JC Kluin PM Noordijk EM Primary extranodal non-Hodgkin's lymphoma (NHL): the impact of alternative definitions tested in the Comprehensive Cancer Centre West population-based NHL registry Ann Oncol 2003 14 1 131 139 10.1093/annonc/mdg004 12488305

[B5] Herrmann R Panahon AM Barcos MP Walsh D Stutzman L Gastrointestinal involvement in non-Hodgkin's lymphoma Cancer 1980 46 1 215 222 10.1002/1097-0142(19800701)46:1<215::AID-CNCR2820460136>3.0.CO;2-6 7388763

[B6] Gospodarowicz MK Ferry JA Cavalli F Mauch PM, Armitago JO, Harris NL Unique aspects of primary extranodal lymphomas Non-Hodgkin's Lymphomas 2003 Philadelphia, PA: Lippincott Williams & Wilkins 685 707

[B7] Wiseman C Liao KT Primary lymphoma of the breast Cancer 1972 29 6 1705 1712 10.1002/1097-0142(197206)29:6<1705::AID-CNCR2820290640>3.0.CO;2-I 4555557

[B8] Cheson BD Horning SJ Coiffier B Shipp MA Fisher RI Connors JM Lister TA Vose J Grillo-Lopez A Hagenbeek A Report of an international workshop to standardize response criteria for non-Hodgkin's lymphomas. NCI Sponsored International Working Group J Clin Oncol 1999 17 4 1244 1253 10561185 10.1200/JCO.1999.17.4.1244

[B9] Kalbfleisch JD Prentice RL The Statistical Analysis of Failure Time Data 1980 New York: John Wiley and Sons

[B10] Gholam D Bibeau F El Weshi A Bosq J Ribrag V Primary breast lymphoma Leuk Lymphoma 2003 44 7 1173 1178 10.1080/1042819031000079195 12916870

[B11] Feugier P Van Hoof A Sebban C Solal-Celigny P Bouabdallah R Ferme C Christian B Lepage E Tilly H Morschhauser F Long-term results of the R-CHOP study in the treatment of elderly patients with diffuse large B-cell lymphoma: a study by the Groupe d'Etude des Lymphomes de l'Adulte J Clin Oncol 2005 23 18 4117 4126 10.1200/JCO.2005.09.131 15867204

[B12] Ryan G Martinelli G Kuper-Hommel M Tsang R Pruneri G Yuen K Roos D Lennard A Devizzi L Crabb S Primary diffuse large B-cell lymphoma of the breast: prognostic factors and outcomes of a study by the International Extranodal Lymphoma Study Group Ann Oncol 2008 19 2 233 241 10.1093/annonc/mdm471 17932394

[B13] Aviles A Delgado S Nambo MJ Neri N Murillo E Cleto S Primary breast lymphoma: results of a controlled clinical trial Oncology 2005 69 3 256 260 10.1159/000088333 16166814

[B14] Au WY Chan AC Chow LW Liang R Lymphoma of the breast in Hong Kong Chinese Hematol Oncol 1997 15 1 33 38 10.1002/(SICI)1099-1069(199702)15:1<33::AID-HON595>3.0.CO;2-K 9378471

[B15] Ha CS Dubey P Goyal LK Hess M Cabanillas F Cox JD Localized primary non-Hodgkin lymphoma of the breast Am J Clin Oncol 1998 21 4 376 380 10.1097/00000421-199808000-00012 9708637

[B16] Jennings WC Baker RS Murray SS Howard CA Parker DE Peabody LF Vice HM Sheehan WW Broughan TA Primary breast lymphoma: the role of mastectomy and the importance of lymph node status Ann Surg 2007 245 5 784 789 10.1097/01.sla.0000254418.90192.59 17457172 PMC1877073

[B17] Wong WW Schild SE Halyard MY Schomberg PJ Primary non-Hodgkin lymphoma of the breast: The Mayo Clinic Experience J Surg Oncol 2002 80 1 19 25 discussion 26 10.1002/jso.10084 11967901

[B18] Zucca E Conconi A Mughal TI Sarris AH Seymour JF Vitolo U Klasa R Ozsahin M Mead GM Gianni MA Patterns of outcome and prognostic factors in primary large-cell lymphoma of the testis in a survey by the International Extranodal Lymphoma Study Group J Clin Oncol 2003 21 1 20 27 10.1200/JCO.2003.11.141 12506165

[B19] Domchek SM Hecht JL Fleming MD Pinkus GS Canellos GP Lymphomas of the breast: primary and secondary involvement Cancer 2002 94 1 6 13 10.1002/cncr.10163 11815954

[B20] Miller TP Dahlberg S Cassady JR Adelstein DJ Spier CM Grogan TM LeBlanc M Carlin S Chase E Fisher RI Chemotherapy alone compared with chemotherapy plus radiotherapy for localized intermediate- and high-grade non-Hodgkin's lymphoma N Engl J Med 1998 339 1 21 26 10.1056/NEJM199807023390104 9647875

[B21] Miller TP LeBlanc M Spier CM Chase E Fischer RJ CHOP alone compared to CHOP plus radiotherapy for early stage aggressive non-Hodgkin's lymphomas: update of the Southwest Oncology Group (SWOG) Randomized Trial Blood 2001 98 742a 743a 10.1182/blood.V98.3.705

[B22] Coiffier B Lepage E Briere J Herbrecht R Tilly H Bouabdallah R Morel P Van Den Neste E Salles G Gaulard P CHOP chemotherapy plus rituximab compared with CHOP alone in elderly patients with diffuse large-B-cell lymphoma N Engl J Med 2002 346 4 235 242 10.1056/NEJMoa011795 11807147

[B23] Pfreundschuh M Trumper L Osterborg A Pettengell R Trneny M Imrie K Ma D Gill D Walewski J Zinzani PL CHOP-like chemotherapy plus rituximab versus CHOP-like chemotherapy alone in young patients with good-prognosis diffuse large-B-cell lymphoma: a randomised controlled trial by the MabThera International Trial (MInT) Group Lancet Oncol 2006 7 5 379 391 10.1016/S1470-2045(06)70664-7 16648042

[B24] Persky DO Unger JM Spier CM Stea B LeBlanc M McCarty MJ Rimsza LM Fisher RI Miller TP Phase II study of rituximab plus three cycles of CHOP and involved-field radiotherapy for patients with limited-stage aggressive B-cell lymphoma: Southwest Oncology Group study 0014 J Clin Oncol 2008 26 14 2258 2263 10.1200/JCO.2007.13.6929 18413640

[B25] Aviles A Castaneda C Neri N Cleto S Nambo MJ Rituximab and dose dense chemotherapy in primary breast lymphoma Haematologica 2007 92 8 1147 1148 10.3324/haematol.10892 17650450

[B26] Rosenwald A Wright G Chan WC Connors JM Campo E Fisher RI Gascoyne RD Muller-Hermelink HK Smeland EB Giltnane JM The use of molecular profiling to predict survival after chemotherapy for diffuse large-B-cell lymphoma N Engl J Med 2002 346 25 1937 1947 10.1056/NEJMoa012914 12075054

[B27] Hans CP Weisenburger DD Greiner TC Gascoyne RD Delabie J Ott G Muller-Hermelink HK Campo E Braziel RM Jaffe ES Confirmation of the molecular classification of diffuse large B-cell lymphoma by immunohistochemistry using a tissue microarray Blood 2004 103 1 275 282 10.1182/blood-2003-05-1545 14504078

[B28] Nyman H Adde M Karjalainen-Lindsberg ML Taskinen M Berglund M Amini RM Blomqvist C Enblad G Leppa S Prognostic impact of immunohistochemically defined germinal center phenotype in diffuse large B-cell lymphoma patients treated with immunochemotherapy Blood 2007 109 11 4930 4935 10.1182/blood-2006-09-047068 17299093

[B29] Winter JN Weller EA Horning SJ Krajewska M Variakojis D Habermann TM Fisher RI Kurtin PJ Macon WR Chhanabhai M Prognostic significance of Bcl-6 protein expression in DLBCL treated with CHOP or R-CHOP: a prospective correlative study Blood 2006 107 11 4207 4213 10.1182/blood-2005-10-4222 16449523 PMC1895783

